# Re-Purposing a Rho-Associated Coiled-Coil Kinase (ROCK) Inhibitor for Alzheimer’s Disease

**DOI:** 10.3390/jcm15093379

**Published:** 2026-04-28

**Authors:** Xavier Cambi, Zhiqing Liu, Kevin Guo, Weiming Xia

**Affiliations:** 1 Department of Pharmacology, Physiology and Biophysics, Chobanian & Avedisian School of Medicine, Boston University, Boston, MA 02118, USA; xcambi@bu.edu; 2 Geriatric Research Education and Clinical Center, VA Bedford Healthcare System, Bedford, MA 01730, USA; zhiqing.liu@va.gov; 3 Avice Laboratories, Acton, MA 01720, USA; kevin.guo@avicelabs.com; 4 Department of Biological Sciences, Kennedy College of Sciences, University of Massachusetts Lowell, Lowell, MA 01854, USA

**Keywords:** Alzheimer, ROCK, pharmacokinetics, efficacy, proteomics

## Abstract

**Background/Objectives:** Currently available treatments approved by the Food and Drug Administration for Alzheimer’s disease (AD) either only target the symptoms of AD or, if disease-modifying, have severe side effects. This study aims to explore the potential of the FDA-approved Rho-associated kinase (ROCK) inhibitor netarsudil to reduce tau, a pathological protein in AD. **Methods:** We explored the pharmacokinetic and pharmacodynamic properties of netarsudil following a single intraperitoneal (i.p.) injection in wild-type mice. The efficacy of netarsudil was assessed using ELISA targeting tau/phosphorylated tau (ptau), as well as mass spectrometry-based proteomics. **Results:** We found that netarsudil is brain permeable, reaches peak concentrations rapidly and has moderate but sustained exposure in the central nervous system (CNS). Additionally, there was a statistically significant negative association between brain netarsudil exposure and tau and phosphorylated tau at residue 181 (ptau181). The exploratory proteomic analysis of mouse brains exposed to netarsudil revealed changes in mitochondrial function, enrichment of metallothioneins Mt1 and Mt2, and suppression of the AD-related genes Pzp and Serpina3m. **Conclusions:** The apparent reduction in AD pathological protein tau/ptau and a neuroprotective proteomic profile in vivo suggest the potential for netarsudil to be developed as a new AD therapeutic agent.

## 1. Introduction

In 2010, there were 4.7 million individuals aged 65 and older with Alzheimer’s disease (AD) in the U.S.; this number rose to 5.8 million in 2020 and is expected to rise to 13.8 million in 2050 [1,2]. This stark increase will lead to a subsequent increase in the burden of care—underscoring the importance of developing novel preventative measures [2]. Current treatments for AD include cognitive enhancers and monoclonal antibodies, but both come with their own sets of risks and benefits [3,4,5]. Moreover, neither of these is a cure for AD; one treats the symptoms, and the other delays the progression of amyloid pathology. A cure for AD has still not been found, so recent AD literature has begun exploring alternative pathways.

AD is characterized by two pathologies: extracellular amyloid beta (Aβ) plaques and intracellular neurofibrillary tangles (NFTs) [6]. Aβ plaque forms when amyloid precursor protein (APP) undergoes amyloidogenic processing and produces Aβ monomers of different lengths into the extracellular space—the main isoforms being Aβ40 and Aβ42. These monomers aggregate to form Aβ plaques, with Aβ42 being the dominant isoform in AD [6,7]. On the other hand, NFTs form when microtubule-associated protein tau is hyperphosphorylated, loses its affinity for microtubules, solubilizes, and aggregates [8]. Tau can be phosphorylated at multiple sites, resulting in several forms of phosphorylated tau (e.g., ptau181) that can be impacted by pharmaceutical intervention [9].

Current treatments are mainly limited to cognitive enhancers and anti-amyloid therapies (AAT). One class of cognitive enhancers is the acetylcholinesterase inhibitors (AChE-I) [3,10]. These drugs are commonly prescribed to AD patients as their cognitive decline coincides with cholinergic and ACh deficiency [10]. However, the effects of these drugs diminish over time and do not address the underlying pathology [10]. The AATs, on the other hand, are disease-modifying drugs that directly target and reduce Aβ load in patients with AD [4]. Currently, there are two FDA-approved AATs in clinical use for the treatment of patients with mild cognitive impairment or mild AD; the first one was lecanemab (leqembi), which received FDA approval in 2023 [11,12]. These drugs are vital for early intervention in AD to delay disease progression. However, treated patients experienced amyloid-related imaging abnormalities (ARIA), specifically ARIA-edema (ARIA-E) and ARIA-hemorrhages (ARIA-H) [4]. The risk of vascular side effects is a roadblock for many potential patients. Therefore, alternative approaches are needed for new AD therapy.

Rho-associated coiled-coil kinase (ROCK) is the downstream effector serine/threonine kinase of Rho-A, a Rho-GTPase [13,14]. The kinase has two isoforms, ROCK 1 and ROCK 2, which are 65% homologous overall and 92% homologous in the kinase domain [13,15]. Typically, this pathway regulates actin-mediated cytoskeleton contractility, cell–cell adhesion dynamics, cell proliferation, and cell survival [13,16,17,18]. In the central nervous system, ROCK1/2 phosphorylates downstream signaling molecules, promoting axonal and dendritic retraction, neuronal death, and synapse loss [17]. While ROCK1 is more ubiquitously expressed, ROCK2 is localized to skeletal muscle and the brain [19]. Additionally, ROCK2 has been implicated in many diseases, including AD, Parkinson’s disease, cancers, and vascular disorders; inhibition of ROCK2 specifically ameliorated some aspects of these conditions [19].

The overactivity of ROCK was reported to be associated with mild cognitive impairment (MCI) and AD; both isoforms of ROCK were elevated in patients with MCI and AD compared to controls [20,21]. The elevation of these kinases is associated with the elevation of AD pathological proteins [14,22]. ROCK can phosphorylate APP at S654 and BACE-1 at T654, thereby promoting amyloidogenic processing and Aβ production [14,19]. Regarding tau pathology, ROCK can activate tau kinases, such as GSK3β, by inhibiting AKT [22,23,24]. Expression of both pathological proteins is reduced by genetic or pharmacological inhibition of ROCK [9,14,22,25,26].

ROCK inhibitor fasudil is currently in clinical trial as an AD therapeutic agent. Fasudil was the first ROCK inhibitor to be approved and is primarily used in Japan and China to treat cerebral vasospasm following subarachnoid hemorrhage. However, it has also been explored in AD to assess the effect of pharmacological ROCK inhibition on AD pathology. Fasudil restored cognitive function, reduced oxidative stress, and neuronal apoptosis in APP/PS1 mice, improved working and spatial memory in an aging rodent model, and reduced the Aβ load in 3xTG-AD mice [27,28,29,30]. Additionally, fasudil reduced brain levels of total tau and ptau in the P301L mouse model and reversed reductions in AKT1 and ptau expression in three-dimensional neurospheroids derived from AD patients [22,26].

In this study, we aimed to investigate the ROCK inhibitor netarsudil, typically prescribed to patients with glaucoma and ocular hypertension, for its ability to reduce the levels of tau, one of the two main pathological proteins seen in AD [31]. Netarsudil was selected for its availability in the U.S. as an FDA-approved agent, its current use as a vasodilator, and its mechanism of action similar to that of fasudil as a ROCK inhibitor [31,32].

## 2. Materials and Methods

### 2.1. In Vivo Study

#### 2.1.1. Mouse Model

Wild-type mice (B6C3F1/J) were purchased from Jackson Labs and housed three per cage on a 12 h light/dark cycle with ad libitum access to food and water. All experimental procedures were performed under a protocol approved by the Institutional Animal Care and Use Committee at the VA Bedford Healthcare System (Animal Welfare Assurance Number D16-00036).

#### 2.1.2. Drug Treatment

To explore the effect of netarsudil in vivo, mice were divided into two experimental groups: (1) “NC”, vehicle-treated mice administered saline and 1% DMSO, (2) “N”, mice treated with netarsudil. N groups were further divided into two dosage groups, “N3” and “N30”, the former receiving 3 mg/kg and the latter receiving 30 mg/kg. Vehicle and netarsudil were administered once by intraperitoneal (i.p.) injection. Mice were euthanized at different time points for the pharmacokinetic study (0.5, 1, 2, 4, 8, and 24 h) or at 4 h after dosing for efficacy evaluation, including proteomic analysis and tau/ptau ELISA. All animals were euthanized by exposing them to CO_2_ inhalation according to the approved protocol. The whole brain and plasma tissue were immediately collected, frozen in liquid nitrogen, and stored at −80 °C for analysis. For the pharmacokinetic study, 30 mice were treated with either vehicle (*n* = 3) or N30 (*n* = 27). At 1, 2, 8, and 24 h, 4 mice were included per group. At 0.5 and 4 h, 6 and 5 mice were included per group, respectively. For the single time point studies, 18 mice were treated with either vehicle (*n* = 6), N3 (*n* = 6), or N30 (*n* = 6).

#### 2.1.3. Determination of Drug Levels in the Brain and Plasma

Established Liquid Chromatography–Mass Spectrometry (LC-MS) methods were adapted to determine netarsudil levels in the brains of mice after a single i.p. injection. The LC-MS system consists of an UltiMate 3000 UHPLC automated system coupled to a TSQ Quantiva triple-quadrupole mass spectrometer (Thermo Fisher, Waltham, MA, USA). Brain tissues were processed using a TissueLyser LT (Qiagen, Valencia, CA, USA) and centrifuged at 17,000× *g* for 20 min at 4 °C. Subsequently, 50 μL of the supernatant was transferred to a new vial and mixed with 130 μL of ice-cold acetonitrile and 20 μL of ranitidine (100 ng/mL), the internal standard solution. The samples were centrifuged at 20,000× *g* for 5 min at 4 °C, and the final supernatants were transferred to HPLC vials for LC-MS analysis. Chromatographic separation was achieved using an ACQUITY UPLC BEH C18 column (75 × 2.1 mm, 1.7 μm particle size; Waters, Milford, MA, USA). The mobile phases consisted of water with 0.1% formic acid (mobile phase A) and acetonitrile with 0.1% formic acid (mobile phase B). A linear gradient elution was employed, starting from 3% to 95% B over 9 min, held at 95% B for 10 min, then returned to 3% B over 1 min, followed by a 5 min equilibration at 3% B. The flow rate was maintained at 0.10 mL/min, and the heated electrospray ionization (H-ESI) source operated in positive ion mode. Data acquisition was carried out in selected reaction monitoring (SRM) mode. The ion spray voltage was set to 3.7 kV, and the ion transfer tube temperature was maintained at 325 °C. The monitored mass-to-charge (*m*/*z*) transitions were 454 → 425 for netarsudil and 315 → 176 for ranitidine, with a second transition for each analyte used for confirmation (Supplementary Figure S1A–E).

#### 2.1.4. Determination of Brain and Blood Pharmacokinetic Parameters

The pharmacokinetic properties of netarsudil were evaluated in plasma and brain following a single i.p. dose of 30 mg/kg in mice. Samples were collected from 0.5 to 24 h following injection, and molar concentrations (µM) were quantified using LC-MS/MS. Since each subject was sampled at a single time point, pharmacokinetic profiles across each tissue were evaluated using a naïve pooled (composite) noncompartmental analysis. Individual molar concentrations at each time point were averaged to obtain the composite brain concentration, and the standard deviation (SD) and number of observations (*n*) were also calculated for each time point. Data were cleaned and organized in RStudio (version 2026.01.0+392) using the tidyverse package. Composite pharmacokinetic parameters were calculated in RStudio using the PKNCA package. Area under the concentration–time curve to the last measurable point (AUC_0–t_) was calculated using the linear-up/log-down trapezoidal method. Maximum concentration (C_max_) and time to maximum concentration (T_max_) were calculated using the composite brain concentrations.

The terminal elimination rate constant (λz) was calculated manually with a log-linear regression analysis of the early terminal phase (0.5 to 4 h) of the concentration–time curve. These time points were selected a priori based on the portion of the curve that shows an approximately log-linear decline. Additionally, a small constant (1 × 10^−6^) was added before the log transformation to avoid a log(0) error. These steps were taken to ensure robustness against minor fluctuations in the terminal phase and ensure an accurate λz estimation. The terminal half-life (t½) was calculated as ln(2)/λz.

Total exposure (AUC_(0–∞)_) was calculated as:

AUC_(0–∞)_ = AUC_(0–t)_ + C_last_/λz

The fraction of AUC extrapolated beyond the last observed time point was calculated to assess the reliability of the terminal-phase estimate and reported as the percentage of total exposure derived from extrapolation (Percent AUC Extrapolated).

Area under the first moment curve (AUMC) was calculated using the linear-up/log-down trapezoidal rule applied to the first moment of the concentration–time curve (t·C vs. time). AUMC_(0–t)_ was obtained from the PKNCA output and extrapolated to infinity using the terminal elimination rate constant. Total AUMC_(0–∞)_ was determined as:

AUMC_(0–∞)_ = AUCM_(0–t)_ + C_last_/λz × T_last_/(1⁄λz)

Mean residence time (MRT) was calculated as:

MRT = AUMC_(0–∞)_/AUC_(0–∞)_

Lastly, brain penetration was evaluated by calculating global brain penetration (K_p_) and time-dependent brain penetration. K_p_ was calculated as the ratio of total brain exposure to total plasma exposure:

K_(p,brain)_ = (AUC_brain_)/(AUC_plasma_)

Time-dependent brain penetration was visualized by plotting the brain-to-plasma concentration ratio at each time point using time-matched samples. Ratios were calculated using mean concentrations at each time point to maintain consistency with the naïve pooled analysis. Graphical visualization of the composite brain concentration–time profile was performed using ggplot2, with a log-scaled y-axis, and SD bars were included to illustrate variability across samples.

#### 2.1.5. Quantification of Tau and Ptau Brain Levels

ELISA was performed to quantify brain levels of tau and phosphorylated tau (ptau) at position 181 using our previously published method [33]. Briefly, sample lysis buffer (2% SDS, 0.5M TEAB, protease/phosphatase inhibitor cocktail; ratio 1:5 per mg of wet tissue) was added to each brain tissue and then homogenized by TissueLyser LT (Qiagen, Valencia, CA, USA). Tissue homogenates were centrifuged at 17,000× *g* for 20 min at 4 °C and diluted 10-fold before being loaded onto ELISA plates coated with the corresponding primary antibodies. Plates were read using the MSD Sector Imager 2400 (Rockville, MD, USA) [33]

#### 2.1.6. Analysis of Brain Proteomics Profile

Brain proteomic profiles were analyzed using our previously published methods [34,35]. Briefly, brain lysates were prepared, and protein concentration was measured using a NanoDrop One (Thermo Fisher). 100 μg of protein from each sample was reduced with tris(2-carboxyethyl)phosphine (TCEP), alkylated with iodoacetamide, precipitated with acetone, and digested with trypsin overnight. Tryptic-digested peptides from brain samples were labeled with TMT 10-plex reagents (Thermo Fisher). The combined TMT-labeled samples were dried and reconstituted in trifluoroacetic acid, then desalted using an Oasis HLB 96-well μElution plate (Waters).

LC-MS/MS analysis was performed on a Q Exactive Orbitrap MS coupled with a Dionex Ultimate 3000 HPLC system equipped with a nano-ES ion source (Thermo Fisher, Waltham, MA, USA) [34]. The TMT-labeled peptides were separated on a C18 reverse-phase capillary column (PepMap, 75 μm × 500 mm, Thermo Fisher, Waltham, MA, USA) with linear gradients of 2–35–90% acetonitrile in 0.1% formic acid at a constant flow rate of 300 nL/min for 320 min. The instrument was operated in positive ion mode with the ESI spray voltage set to 1.5 kV. Twenty peptide ions showing the most intense signal from each scan were selected for higher energy collision-induced dissociation (HCD)-MS/MS analysis (normalized collision energy 32) in the Orbitrap. The data were acquired using Thermo Xcalibur 3.1.6.10.

Raw data were processed using Proteome Discoverer (Version 3.1, Thermo Fisher Scientific, Waltham, MA, USA). Data were searched against the Mus musculus Universal Protein Resource (UniProt) sequence database. The search parameters were: trypsin digestion with two missed cleavages allowed; static modification: carbamidomethyl of cysteine C-terminus and TMT 10-plex (peptide labeled) for N-terminus and lysine; dynamic modification: oxidation of methionine, acetylation of N-terminus, demethylation of methionine, and acetylation and demethylation of methionine; MS tolerance: 20 ppm and 0.05 Da; false discovery rate (FDR) of less than 0.01 at peptide and protein levels; and required peptide length: 6 ≤ X ≤ 144. Reporter parameters were automatic reporter abundances, and Quan value corrections were applied.

The grouped abundances of proteins in brains from netarsudil or vehicle-treated animals (*n* = 3 for netarsudil and *n* = 2 for vehicle) were analyzed in RStudio (version 2026.01.0+392) using the limma package. Proteins with >50% missing values were excluded, and those with zero intensities were treated as missing. The data were log2-transformed and normalized across channels using quantile normalization. Differential expression was assessed using empirical Bayes-moderated linear modeling with mean–variance trend correction (trend = TRUE).

Principal component analysis was performed on normalized expression values. Heatmaps were generated using row-scaled (z-score) expression values for all nominally significant proteins (*n* = 31) or the top 20 proteins ranked by raw *p*-value. Nominal significance was defined as *p*-values less than 0.05 combined with a minimum fold-change threshold of 1.3. Adjusted *p*-values (Benjamini–Hochberg FDR) are reported for transparency but were not used to define nominal significance. Enrichment significance was assessed using FDR-adjusted q-values (<0.05). Heatmaps and volcano plots were generated using pheatmap and ggplot2.

### 2.2. Statistical Analysis

Linear regression analysis was used to assess for a correlation between brain levels of netarsudil and brain levels of tau and ptau. Additionally, a one-way ANOVA with treatment as a between-subjects factor was used to analyze the effect of netarsudil dosage on brain netarsudil levels. Tukey’s post hoc tests were performed after significant main and interaction effects were observed in the ANOVA. The significance level (α) was set at 0.05. Data analysis and graph processing were performed using Prism 11.0.0 (GraphPad Software LLC, San Diego, CA, USA).

## 3. Results

### 3.1. Netarsudil Penetrates the BBB and Exhibits Prolonged Brain Presence Compared with Systemic Circulation

**Figure 1:**
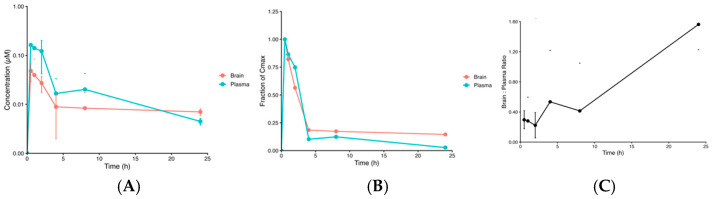
Plasma and brain pharmacokinetics of netarsudil. (**A**) Plasma (*n* = 2) and brain (*n* = 2) concentrations of netarsudil in N30-treated mice were measured over 24 h following a single intraperitoneal dose in wild-type mice. Concentrations are plotted on a logarithmic scale to highlight the terminal elimination phase. Each point represents the mean of all animals sampled at that time point (*n* = 2). Error bars denote standard deviation (SD). (**B**) Tissue concentrations of netarsudil were normalized to their respective Cmax. Each point represents the mean normalized concentration (*n* = 2). These concentration–time profiles demonstrate that netarsudil undergoes rapid initial distribution in both matrices but exhibits prolonged residence in the brain relative to plasma. (**C**) Time-dependent illustration of the brain-to-plasma ratio of netarsudil. Brain-to-plasma ratios were less than 1 between 0.5 and 8 h, then increased to 1.56 at 24 h post-dose. This implies slower apparent clearance from the brain relative to plasma. Brain-to-plasma ratios were calculated using mean concentrations at each time point (*n* = 2). Error bars denote SD.

Following i.p. administration of netarsudil, measurable concentrations of the compound were detected in both plasma and brain up to 24 h post-injection. Plasma concentrations increased rapidly, reaching a Cmax of 0.163 µM at 0.5 h (Tmax), and subsequently declined (Figure 1A). Brain concentrations also peaked at 0.5 h, with a Cmax of 0.0483 µM, indicating rapid distribution into the central nervous system (Figure 1A).

Noncompartmental analysis demonstrated a total plasma exposure AUC_(0–∞)_ of 0.619 µM·h and an AUMC_(0–∞)_ of 3.50 µM·h^2^ (Table 1). The terminal half-life and mean residence time (MRT) were 1.05 and 5.65 h, respectively. In brain, total exposure AUC_(0–∞)_ was 0.257 µM·h, with AUMC_(0–∞)_ of 2.41 µM·h^2^. The terminal half-life and MRT were 1.43 and 8.77 h, respectively. The percentages extrapolated AUC for plasma and brain were 4.06% and 6.66%, respectively, indicating adequate characterization of the terminal elimination phase.

Normalized concentration–time curves demonstrated similar early distribution kinetics in plasma and brain, followed by a more prolonged persistence in brain tissue (Figure 1B). Time-matched brain-to-plasma ratios were less than 1 at early time points, reflecting higher plasma exposure initially, as expected (Figure 1C). Ratios increased at later time points, exceeded 1, and peaked at 1.56 at 24 h. This indicates slower apparent brain clearance relative to plasma, suggesting sustained CNS exposure. These results suggest that netarsudil penetrates the brain and exhibits moderate, prolonged residence in the brain compared with the systemic circulation.

### 3.2. Netarsudil Is Brain Permeable

**Figure 2:**
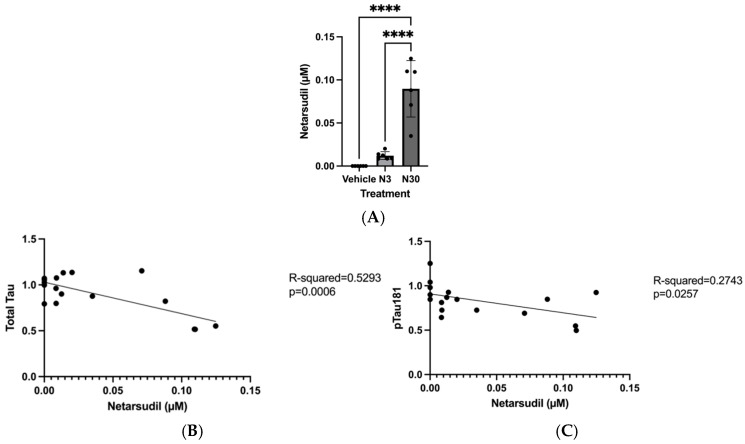
Increasing brain netarsudil exposure is associated with decreased brain levels of total tau and ptau181. Mice (5 to 6 weeks old) were divided into independent groups and given vehicle (*n* = 6), 3 mg/kg (N3, *n* = 6), or 30 mg/kg (N30, *n* = 6) and were sacrificed after 4 h. Brain tissue was collected and underwent ELISA analysis to quantify tau and ptau181, and LC-MS/MS to quantify drug levels. (**A**) One-way ANOVA revealed that mice treated with netarsudil had significantly higher brain levels of netarsudil compared to vehicle (F(2,15) = 7.657, *p* = 0.0001, 95% C.I. = [−0.1183,−0.06104]). Data is presented as mean ± SD, **** *p* < 0.0001. (**B**) Linear regression analysis demonstrated a negative association between brain levels of netarsudil and total tau (R^2^ = 0.5293, F(1,16) = 17.99, *p* = 0.0006). (**C**) Linear regression analysis demonstrated a negative association between brain levels of netarsudil and ptau181 (R^2^ = 0.2743, F(1,16) = 6.046, *p* = 0.0257).

The mean brain levels of netarsudil reflected different dosages of netarsudil (3 and 30 mg/kg) delivered to mice (Figure 2A). The one-way ANOVA revealed a statistically significant difference in brain netarsudil levels among treatment groups (F(2,15) = 7.657, *p* = 0.0001). Tukey’s HSD Test for multiple comparisons found that the average brain levels of netarsudil were significantly higher in mice treated with 30 mg/kg netarsudil (N30) than in mice treated with 3 mg/kg netarsudil (N3) (*p* < 0.0001) or vehicle (*p* < 0.0001).

### 3.3. Brain Levels of Netarsudil Were Significantly Associated with Brain Levels of Total Tau and ptau181

By administering two doses of netarsudil (3 and 30 mg/kg) to mice, we observed a dose-dependent relationship between netarsudil and tau levels in individual mice’s brains. We quantified levels of netarsudil using LC-MS and tau and ptau181 using ELISA, and we found that the association between brain levels of netarsudil and total tau in wild-type mice was statistically significant (R^2^ = 0.5293, F(1,16) = 17.99, *p* = 0.0006) (Figure 2B). Quantification of ptau181 in the same brain lysates further indicated a significant association between brain netarsudil levels and ptau181. The overall regression was statistically significant (R^2^ = 0.2743, F(1,16) = 6.046, *p* = 0.0257) (Figure 2C). Therefore, brain netarsudil exposure was negatively associated with levels of tau and ptau181 in the brains of wild-type mice.

### 3.4. Acute Treatment with Netarsudil at 30 mg/kg Causes Proteome-Wide Changes

The LC/MS analysis was designed to quantify abundances for over 2440 proteins from our mouse brains exposed to netarsudil (or vehicle), and 2306 proteins had quantifiable abundances suitable for analysis. Of these proteins, the LIMMA analysis identified 31 that met the significance criteria for netarsudil treatment (*p* < 0.05 and log2 fold change > 1.3) (Supplementary Table S1). None of these proteins met the significance criteria after Benjamini–Hochberg multiple testing correction. Therefore, this proteomic study was conducted as an exploratory study.

Under the described threshold for nominal significance, 31 proteins were identified as differentially expressed between the netarsudil- and vehicle-treated groups (Supplementary Table S1). As stated above, none of these proteins remained significant following multiple testing correction with the Benjamini–Hochberg method (FDR < 0.05) (Supplementary Table S1).

The protein with the strongest statistical signal was metallothionein-1 (Mt-1) and exhibited the largest increase in the netarsudil-treated group (log_2_FC = 1.18, *p* = 8.8 × 10^−5^) (Supplementary Table S1). Additional proteins with increased abundances in the compound-treated group included: Camk2 (log_2_FC = 0.38, *p* = 0.011), metallothionein 2 (Mt-2) (log_2_FC = 0.56, *p* = 0.013), and Tanc1 (log_2_FC = 0.95, *p* = 0.021) (Supplementary Table S1). Proteins with strong statistical signals with decreased abundance in the netarsudil-treated group included: Tars3 (log_2_FC = −0.54, *p* = 1.4 × 10^−3^), Fastdk1 (log_2_FC = −1.32, *p* = 1.3 × 10^−3^), Tars3 (log_2_FC = −0.54, *p* = 1.4 × 10^−3^), and Pzp (log_2_FC = −0.61, *p* = 5.6 × 10^−3^) (Supplementary Table S1). If the proteomic analysis is considered exploratory, the unadjusted *p*-values and fold changes offer an opportunity to generate hypotheses about netarsudil and its potential to promote neuroprotective pathways.

**Figure 3:**
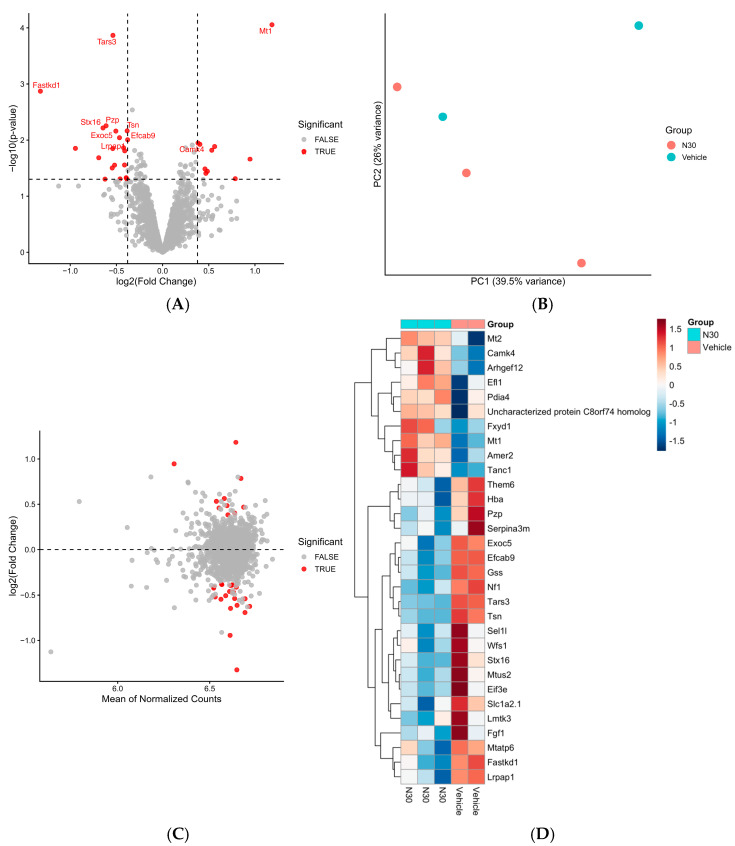
The proteomic analysis of wildtype mice treated with Netarsudil at 30 mg/kg. (**A**) Volcano plot of the differential protein abundances following treatment at 30 mg/kg netarsudil (*n* = 3) or vehicle (*n* = 2) using LIMMA empirical Bayes-moderated linear modeling. The x-axis represents the log2 fold change, and the y-axis represents the −log10 (raw *p*-values). The proteins that met nominal significance criteria (*p* < 0.05 and absolute log_2_FC > 0.38) are highlighted in red. The 10 proteins with the lowest raw *p*-value are labeled. (**B**) The principal component analysis (PCA) of normalized log2 protein abundances from netarsudil or vehicle-treated mice. Each point represents an individual sample, and the first two principal components (PC1 and PC2) are shown; they capture the largest sources of variance. Separation in treatment groups is used to indicate differences in protein expression profiles. (**C**) The MA plot of the normalized proteomic data comparing netarsudil- and vehicle-treated mice. The x-axis shows the average normalized expression, and the y-axis shows the log2 fold change. Proteins meeting nominal expression criteria are colored red. (**D**) Heat map visualizing all 31 proteins that meet nominal significance criteria. Expression values represent row-scaled (z-score) log_2_-normalized abundances across samples. Hierarchical clustering was performed using Euclidean distance and complete linkage to visualize relative protein expression patterns between netarsudil-treated and vehicle-treated samples.

The change in expression and statistical significance of each protein was further illustrated in a volcano plot, with statistically significant proteins colored red (Figure 3A). The 10 proteins with the lowest p-score include: Fastkd1, Tars3, Mt1, Stx16, Pzp, Exoc5, Lrpap1, Tsn, Efcab9, and Camk4. Principal Component Analysis (PCA) was performed to understand expression profiles between groups and to visualize the relationship between protein expression and fold changes (Figure 3B,C). Lastly, normalized protein expressions across groups and subjects were visualized using heatmaps (Figure 3D). This demonstrated clear group-level clustering with subjects displaying similar changes in their protein expression profiles.

## 4. Discussion

This study explored the pharmacokinetic profile of the ROCK inhibitor netarsudil, its effects on tau pathology and the brain proteomic profile in wild-type mice. Netarsudil penetrated the blood–brain barrier (BBB) and was detectable in the brain and plasma starting at 0.5 h. Additionally, brain netarsudil exposure was negatively associated with brain levels of total tau and ptau181. Lastly, our exploratory brain proteomic study revealed enrichment in neuroprotective pathways. Since all these experiments were performed using wild-type mice, these results should be interpreted as evidence of target engagement rather than therapeutic efficacy.

Nevertheless, our pharmacokinetic data demonstrate that netarsudil reaches maximum concentration in the brain and blood at 0.5 h. These findings indicate that netarsudil was rapidly absorbed systemically and penetrated the CNS. However, plasma pharmacokinetics showed rapid elimination, with a terminal half-life and MRT of 1.05 and 5.65 h, respectively. On the other hand, brain pharmacokinetics demonstrated prolonged exposure, with a half-life and MRT of 1.43 and 8.77 h, respectively.

While peak brain concentrations were lower than plasma concentrations (0.048 µM vs. 0.163 µM), the equivalent T_max_ values in both tissues suggest rapid penetration across the blood–brain barrier (BBB) following systemic distribution. The global brain-to-plasma exposure (K_p,brain_) was 0.44, indicating lower brain exposure relative to systemic exposure. However, the brain-to-plasma ratios show sustained retention of netarsudil in the CNS. This is consistent with the prolonged residence of netarsudil in the brain following systemic clearance, as indicated by the half-life and MRT.

Since the fraction of the extrapolated AUC was 4.1% and 6.7% for the plasma and brain, it can be concluded that the terminal elimination phase was adequately characterized within the 24 h sampling window. This supports the reliability of the estimated half-life and MRT values and solidifies the conclusion that netarsudil can penetrate the BBB and exhibit sustained CNS exposure following intraperitoneal administration. Therefore, any changes observed in AD biomarker expression and the brain proteome can be attributed to netarsudil administration and brain exposure to netarsudil.

Brain levels of ptau181 species were quantified in this study. Tau can be phosphorylated at several sites, resulting in several forms of phosphorylated tau (e.g., ptau181, 202, and 396)—each associated with different tauopathies and neurodegenerative conditions [33]. Understanding the effect of netarsudil on phosphorylation of tau is critical, especially in the context of previous reports using ROCK inhibitor fasudil. Past literature has established that fasudil reduced brain levels of tau and ptau in P301L mouse models and in three-dimensional neurospheroids derived from AD patients [9,22,26]. Similarly, we found a negative association between brain exposure to netarsudil and levels of tau and ptau during acute netarsudil treatment. Additionally, exploratory proteomic studies reveal that chronic treatment with fasudil activates proteins that mediate the mitochondrial tricarboxylic acid (TCA) cycle [9]. Based on proteomic profiles from our study with netarsudil, we found changes in proteins involved in mitochondrial function such as FAST Kinase Domains 1 (Fastkd1); Fastkd1 is located in mitochondrion and nucleoplasm and involved in mitochondrial RNA metabolic process and regulation of mitochondrial mRNA stability, and it is associated with open-angle glaucoma 1 disease, consistent with the FDA-approved indication of netarsudil [31].

Our exploratory proteomic profiling of changes in metallothioneins (MT, e.g., Mt1, Mt2) supports our future research to test the hypothesis that Mt1 and Mt2 are directly involved in mediating the impact of netarsudil. MTs play an important role in regulating the cellular metabolism of essential metals such as zinc and copper [36]. In the central nervous system, these proteins are synthesized in response to a variety of stimuli, including injury, reactive oxygen species (ROS), metal ions, elevated glucocorticoid levels, and viral infection [36,37,38]. By binding to and sequestering heavy metals, MTs play a protective role against oxidative stress and neuroinflammation—two key players in the pathogenesis of AD [36,37,38,39,40]. Oxidative stress in AD is primarily thought to be caused by Aβ-induced expression of ROS [36,41]. This stress incites a positive feedback loop where the ROS promote Aβ production, and the resulting Aβ further promote oxidative stress [42,43]. Moreover, both Aβ and ROS contribute to the neuroinflammation seen in AD [44,45,46]. In response to Aβ plaques, microglia and astrocytes cluster around the lesions and overexpress MTs [47,48]. This expression is thought to be induced by the upregulation of inflammatory cytokines, free radicals, and metal ions as a result of AD [36,49,50]. Moreover, the upregulation of Mt-1/Mt-2 attenuates Aβ-induced neurotoxicity and neuroinflammatory responses [47]. Our exploratory proteomic analysis revealed enrichment of the neuroprotective Mt1 and Mt2 in response to ROCK inhibition in wild-type brains. This underscores the potential of netarsudil as a treatment for AD, especially if the findings are replicated in animal models.

In addition to changes in metallothionine signaling, we also found that netarsudil affected the expression of genes involved in neuronal growth and integrity. Tanc1 is involved in synaptic signaling and dendritic spine maintenance [51], and was upregulated in wild-type mice treated with netarsudil. On the other hand, Serpina3m and PZP were downregulated in the brains of wild-type mice in response to treatment with netarsudil. Serpina3 and PZP regulate inflammation and are implicated in AD [52,53]. For instance, elevated levels of Serpina3 were observed in the blood, brain, and plasma of AD patients and *Serpina3* polymorphisms were associated with earlier AD onset [52,54,55]. Additionally, murine orthologue *Serpina3n* transcripts and protein levels were elevated in an APP23 AD mouse model [54]. PZP was upregulated in the serum of patients who later developed AD [53]. 

Changes in ROCK’s downstream signaling pathways are not clear. Prior literature demonstrates that ROCK inhibitors suppress these pathways across multiple AD and non-AD models. A single 3 µM dose of fasudil reduced the increases in ptau 202, 231, and 396 seen in 3D neurospheroids generated from AD patients, relative to healthy controls [26]. This occurred concurrently with increased intracellular clusterin and the reversal of the inhibition of AKT expression [26]. Additionally, M1C cells treated with 1 or 10 µM of ROCK inhibitor H-1152 for four days showed a reduction in levels of ptau (PHF-1, CP13, AT180, T270), a downregulation of phosphorylated tau kinase Cdk5, and an upregulation of phosphorylated AKT [22]. Cells treated with 10 µM of H-1152 showed a downregulation of tau kinase GSK3β [22]. The reductions in total tau and ptau were replicated in P301L tauopathy mouse models treated with fasudil at 12 mg/kg/day [22]. It appears that ROCK inhibitors reduce levels of ptau by targeting tau kinase pathways, particularly the AKT/GSK3β pathway.

While this study offers evidence for the potential use of netarsudil in treating AD patients, several limitations should be considered when interpreting these findings. First, the pharmacokinetic study limited data analysis to a 24 h sampling window at six time points (0.5, 1, 2, 4, 8, and 24 h), which could have restricted our ability to characterize brain pharmacokinetic parameters, including the complete elimination of netarsudil in the brain and plasma. Future studies should incorporate more sampling intervals within the 24 h period or extend the sampling window to 36 and 48 h. The second limitation of our efficacy studies was the use of a wild-type model rather than an AD model. Therefore, despite our goal of testing the efficacy of a commercially available, FDA-approved drug against AD pathological proteins, these results should be interpreted as evidence of target engagement rather than therapeutic efficacy. Further validation using an animal model of AD, such as the APP/PS1 model, would be required to support netarsudil efficacy against AD biomarkers. On a similar note, the small group size (*n* = 6) and acute treatment only allow us to conclude that brain netarsudil exposure correlates with reduced tau biomarker levels, rather than a direct reduction in tau synthesis or increase in tau degradation. However, the potential for netarsudil to be efficacious is supported by the efficacy of fasudil. This ROCK inhibitor has already been investigated as a potential AD therapeutic agent in animal models. Third, our proteomic analysis identified 31 significant proteins, but none remained significant after Benjamini–Hochberg correction. Therefore, all pathway-level changes must be labeled as exploratory. Fourth, our ELISA- and MS-based protein quantification and proteomic analyses provide evidence supporting the repurposing of netarsudil as an AD drug; future studies are needed to demonstrate that netarsudil reduces tauopathy, enriches neuroprotective pathways, and suppresses AD-associated pathways in animal models. To illustrate that the neuroprotective properties of ROCK inhibition via netarsudil treatment are adequately demonstrated, future studies would benefit from testing the efficacy of netarsudil following chronic treatment in animal models of AD. Moreover, since the endpoint would be the test of ROCK inhibition in human trials, it would be important to determine the pharmacokinetic parameters of netarsudil following oral dosing.

## 5. Conclusions

Netarsudil is a ROCK inhibitor that demonstrates rapid absorption into the brain. The negative association between brain levels of netarsudil and AD pathological proteins tau and ptau181, a perceived enrichment in metallothionein proteins and neuronal growth pathways and a perceived suppression of AD-related pathways, provide evidence to support future exploration of netarsudil as a new AD therapeutic agent.

## Figures and Tables

**Table 1:** The plasma and brain pharmacokinetics of netarsudil.

Parameter	Plasma	Brain
Cmax (µM)	0.16	0.05
Tmax (h)	0.50	0.50
AUC_0–t_ (µM*h)	0.59	0.26
AUC_0–∞_ (µM*h)	0.62	0.26
AUMC_0–t_ (µM*h^2^)	3.36	2.30
AUMC_0–∞_ (µM*h^2^)	3.50	2.41
t_½_ (h)	1.05	1.43
MRT (h)	5.65	8.77
Percent AUC Extrapolated (%)	4.06	6.66

## Data Availability

Data are available upon submitting a request to the corresponding author.

## Supplementary Materials

The following supporting information can be downloaded at: https://www.mdpi.com/article/10.3390/jcm15093379/s1, Figure S1: Representative LC–MS/MS chromatograms showing the detection of the internal standard and the analyte netarsudil; Table S1: Nominally significant proteins following treatment with netarsudil.

## Abbreviations

The following abbreviations are used in this manuscript:

AAT	anti-amyloid therapies
AD	Alzheimer’s disease
Ab	amyloid beta
AUC	Area under the curve
ARIA	amyloid-related imaging abnormalities
BBB	Blood–brain barrier
FDA	Food and Drug Administration
MRT	Mean residence time
MS	Mass spectrometry
Mt	metallothioneins
NFT	neurofibrillary tangles
ROCK	Rho-associated coiled-coil kinase
ROS	reactive oxygen species
