# Supplementary material for: Re-Purposing a Rho-Associated Coiled-Coil Kinase (ROCK) Inhibitor for Alzheimer’s Disease
Source: J Clin Med. 2026 Apr 28;15(9):3379. doi: 10.3390/jcm15093379 (PMC13164517; doi:10.3390/jcm15093379)
Supplement: Supplementary file 1 [file jcm-15-03379-s001.zip › jcm-4245868-supplementary.pdf]

Supplementary Table S1. Nominally significant proteins following treatment with netarsudil

Protein	$Log_2(FC)$	t-statistic	p-value	Adjusted p-value
Mt1	1.18401492	8.12590182	8.80E-05	0.156637674
Tars3	-0.5377151	-7.5843559	0.00013591	0.156637674
Fastkd1	-1.3229513	-5.1703331	0.0013434	0.995983758
Pzp	-0.6120248	-3.9623132	0.00557843	0.995983758
Stx16	-0.6451795	-3.8933282	0.00609052	0.995983758
Tsn	-0.3837462	-3.800955	0.0068583	0.995983758
Exoc5	-0.5061562	-3.7939805	0.00692042	0.995983758
Lrpap1	-0.4666903	-3.5861122	0.0090842	0.995983758
Efcab9	-0.378652	-3.523273	0.00987535	0.995983758
Camk4	0.38364009	3.42402909	0.01128072	0.995983758
Arhgef12	0.40386247	3.38475142	0.01189532	0.995983758
Mt2	0.56334531	3.3181232	0.01302195	0.995983758
Gss	-0.4225158	-3.282215	0.01367628	0.995983758
Mtus2	-0.9437302	-3.2636568	0.01402821	0.995983758
Them6	-0.5398002	-3.2579042	0.01413927	0.995983758
Amer2	0.53252373	3.20818207	0.01513943	0.995983758
Nf1	-0.4099485	-3.1896696	0.01553095	0.995983758
Hba	-0.6911979	-2.9869115	0.02060236	0.995983758
Tanc1	0.94640528	2.94504403	0.02185425	0.995983758
Eif3e	-0.412112	-2.771826	0.02795498	0.995983758
Slc1a2.1	-0.519922	-2.7682347	0.02809901	0.995983758
Mtatp6	-0.5453017	-2.6871805	0.03156542	0.995983758
Efl1	0.45895249	2.6591503	0.03286598	0.995983758
Pdia4	0.48498147	2.59295678	0.03616407	0.995983758
Fxyd1	0.46846174	2.53627284	0.03926158	0.995983758
Sel1l	-0.3940272	-2.4127981	0.04699615	0.995983758
Wfs1	-0.3902234	-2.3901337	0.04857825	0.995983758
Uncharacterized protein C8orf74 homolog	0.78656227	2.3846885	0.04896643	0.995983758
Lmtk3	-0.4604067	-2.379818	0.04931633	0.995983758
Serpina3m	-0.6233965	-2.3732205	0.04979439	0.995983758
Fgf1	-0.3847035	-2.3704562	0.0499961	0.995983758

Proteins meeting nominal significance criteria, defined as waw $p < 0.05$ and the absolute log2 fold change ≥ 1.3. Adjusted p-values are reported for transparency but were not used to define significance.

Supplementary Figure S1

A.

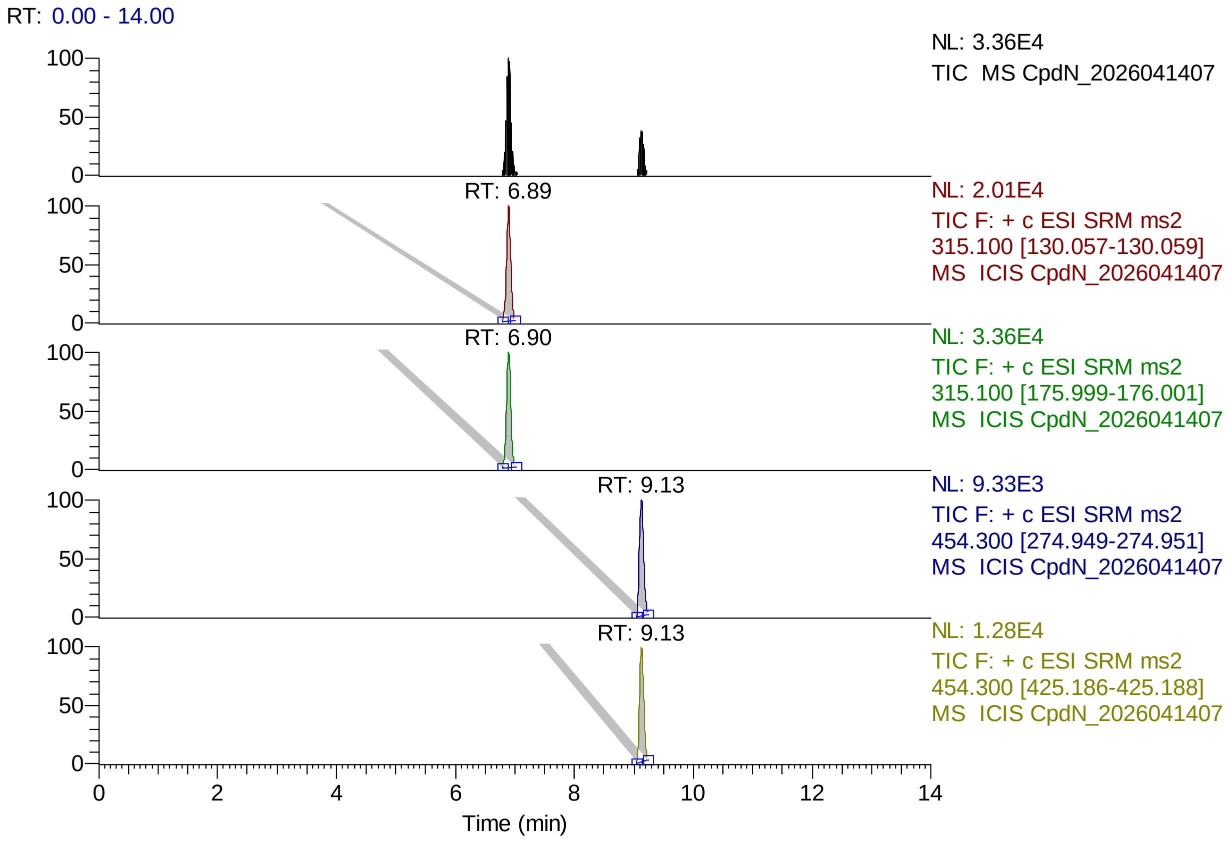

B.

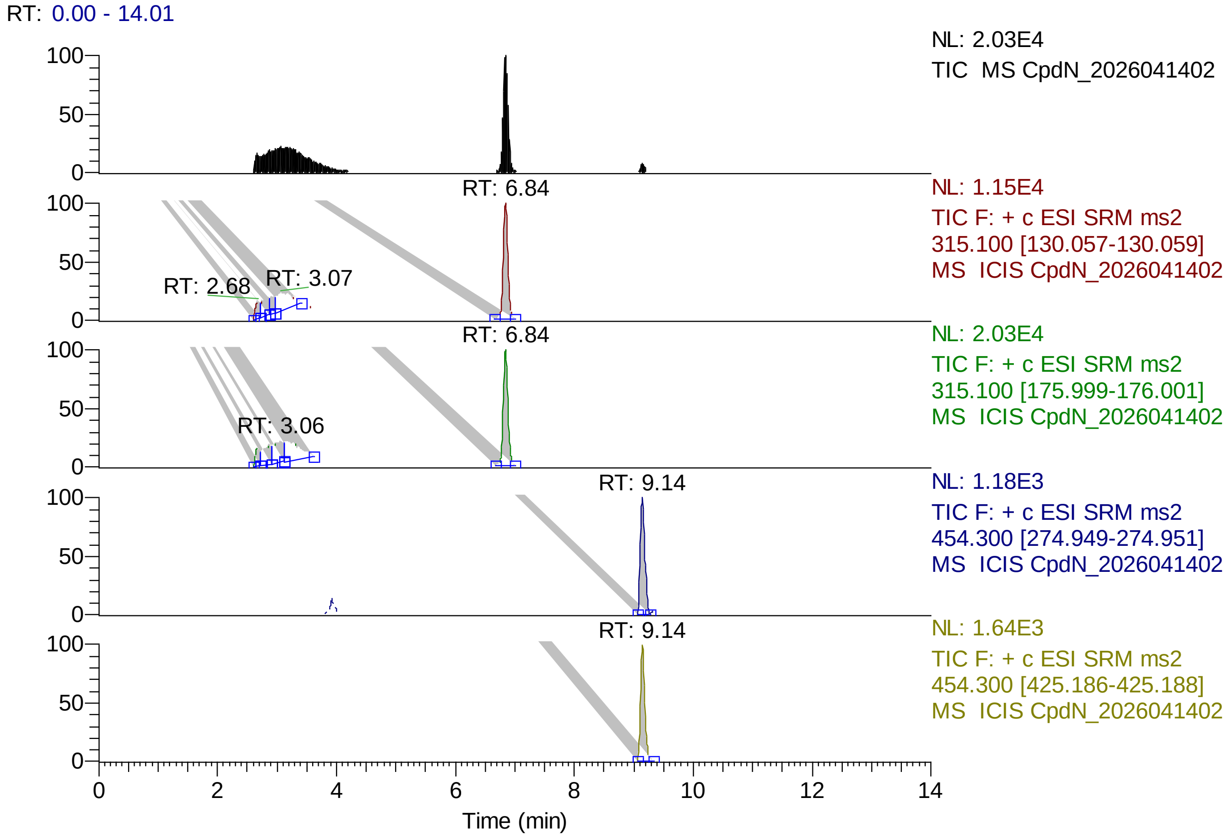

RT: 0.00 - 14.01
NL: 2.03E4
TIC MS CpdN_2026041402
RT: 6.84
RT: 2.68
RT: 3.07
NL: 1.15E4
TIC F: + c ESI SRM ms2 315.100 [130.057-130.059] MS ICIS CpdN_2026041402
RT: 6.84
RT: 3.06
NL: 2.03E4
TIC F: + c ESI SRM ms2 315.100 [175.999-176.001] MS ICIS CpdN_2026041402
RT: 9.14
NL: 1.18E3
TIC F: + c ESI SRM ms2 454.300 [274.949-274.951] MS ICIS CpdN_2026041402
RT: 9.14
NL: 1.64E3
TIC F: + c ESI SRM ms2 454.300 [425.186-425.188] MS ICIS CpdN_2026041402
100
50
0
0
2
4
6
8
10
12
14
Time (min)

C.

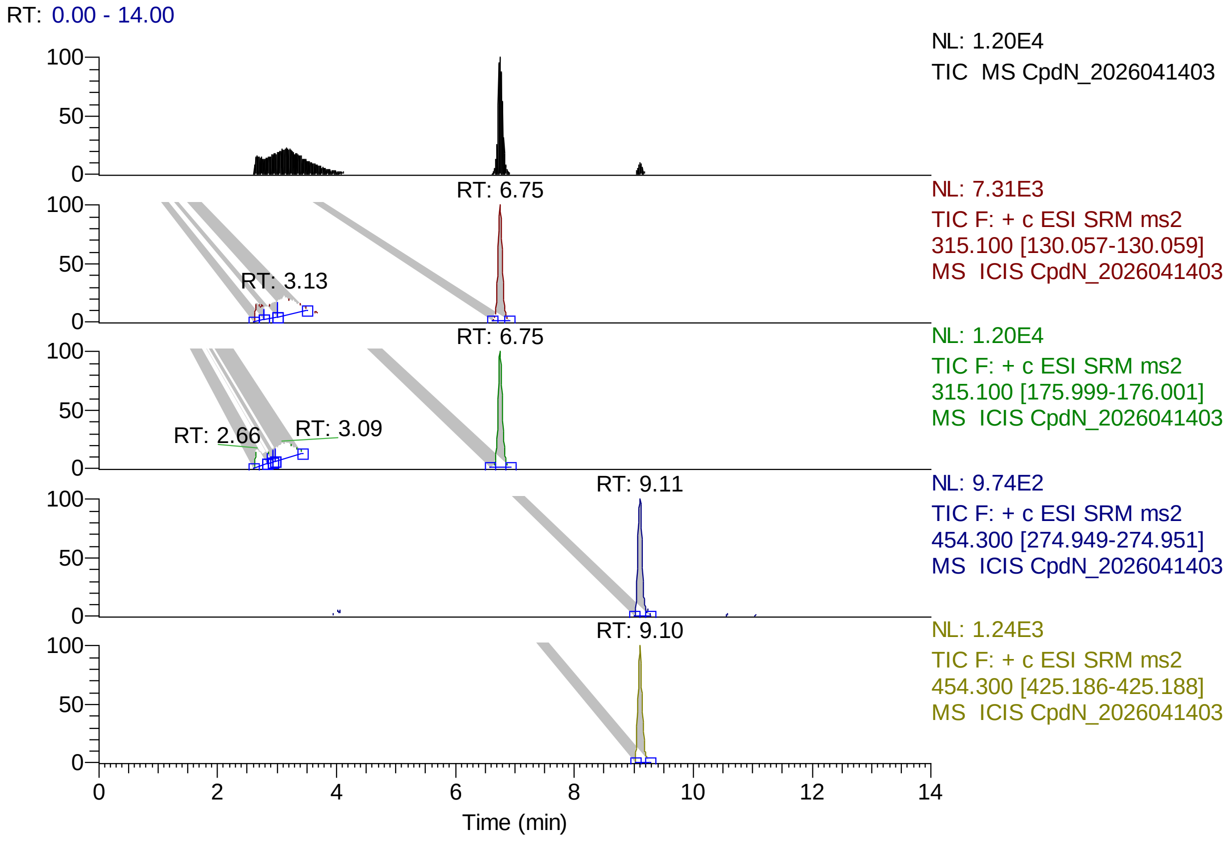

RT: 0.00 - 14.00
NL: 1.20E4
TIC MS CpdN_2026041403
RT: 6.75
RT: 3.13
NL: 7.31E3
TIC F: + c ESI SRM ms2 315.100 [130.057-130.059] MS ICIS CpdN_2026041403
RT: 6.75
RT: 2.66
RT: 3.09
NL: 1.20E4
TIC F: + c ESI SRM ms2 315.100 [175.999-176.001] MS ICIS CpdN_2026041403
RT: 9.11
NL: 9.74E2
TIC F: + c ESI SRM ms2 454.300 [274.949-274.951] MS ICIS CpdN_2026041403
RT: 9.10
NL: 1.24E3
TIC F: + c ESI SRM ms2 454.300 [425.186-425.188] MS ICIS CpdN_2026041403
100
50
0
0
2
4
6
8
10
12
14
Time (min)

D.

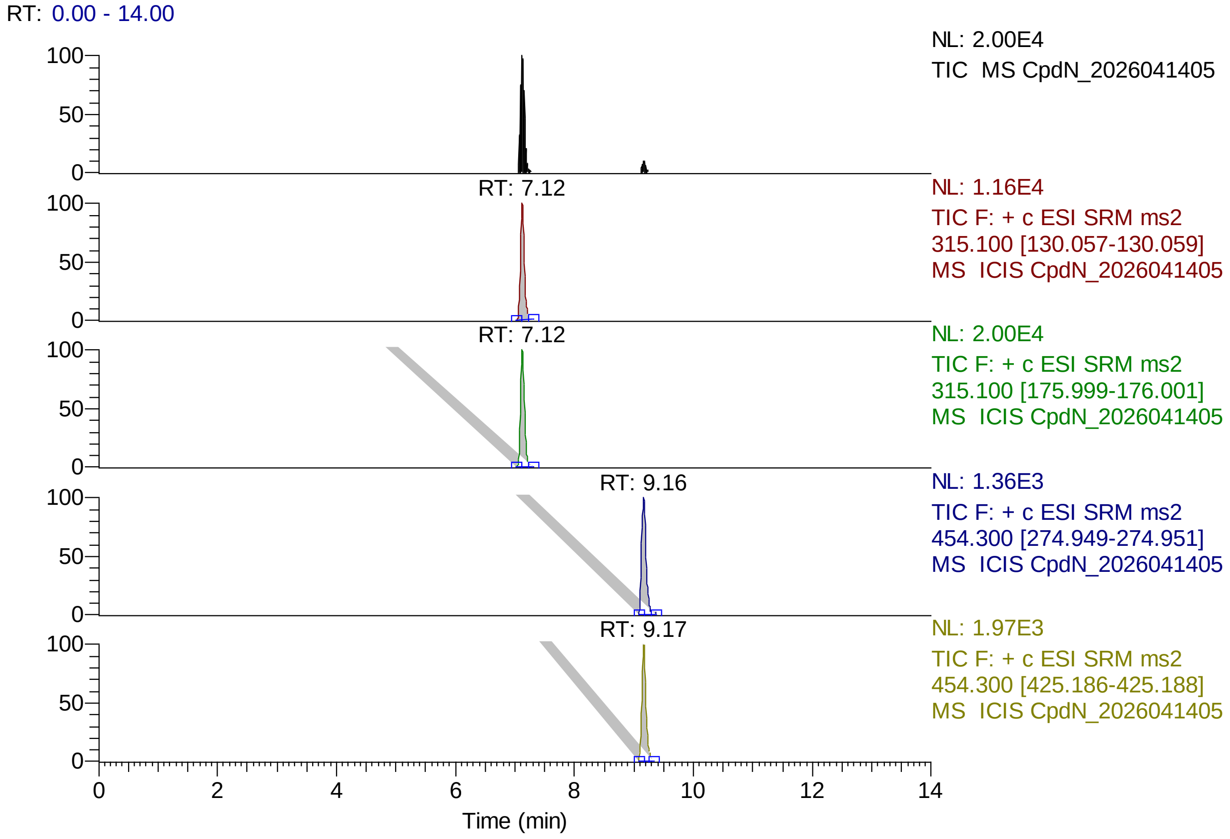
RT: 0.00 - 14.00
NL: 2.00E4
TIC MS CpdN_2026041405
RT: 7.12
NL: 1.16E4
TIC F: + c ESI SRM ms2 315.100 [130.057-130.059] MS ICIS CpdN_2026041405
RT: 7.12
NL: 2.00E4
TIC F: + c ESI SRM ms2 315.100 [175.999-176.001] MS ICIS CpdN_2026041405
RT: 9.16
NL: 1.36E3
TIC F: + c ESI SRM ms2 454.300 [274.949-274.951] MS ICIS CpdN_2026041405
RT: 9.17
NL: 1.97E3
TIC F: + c ESI SRM ms2 454.300 [425.186-425.188] MS ICIS CpdN_2026041405
Time (min)

E.

.

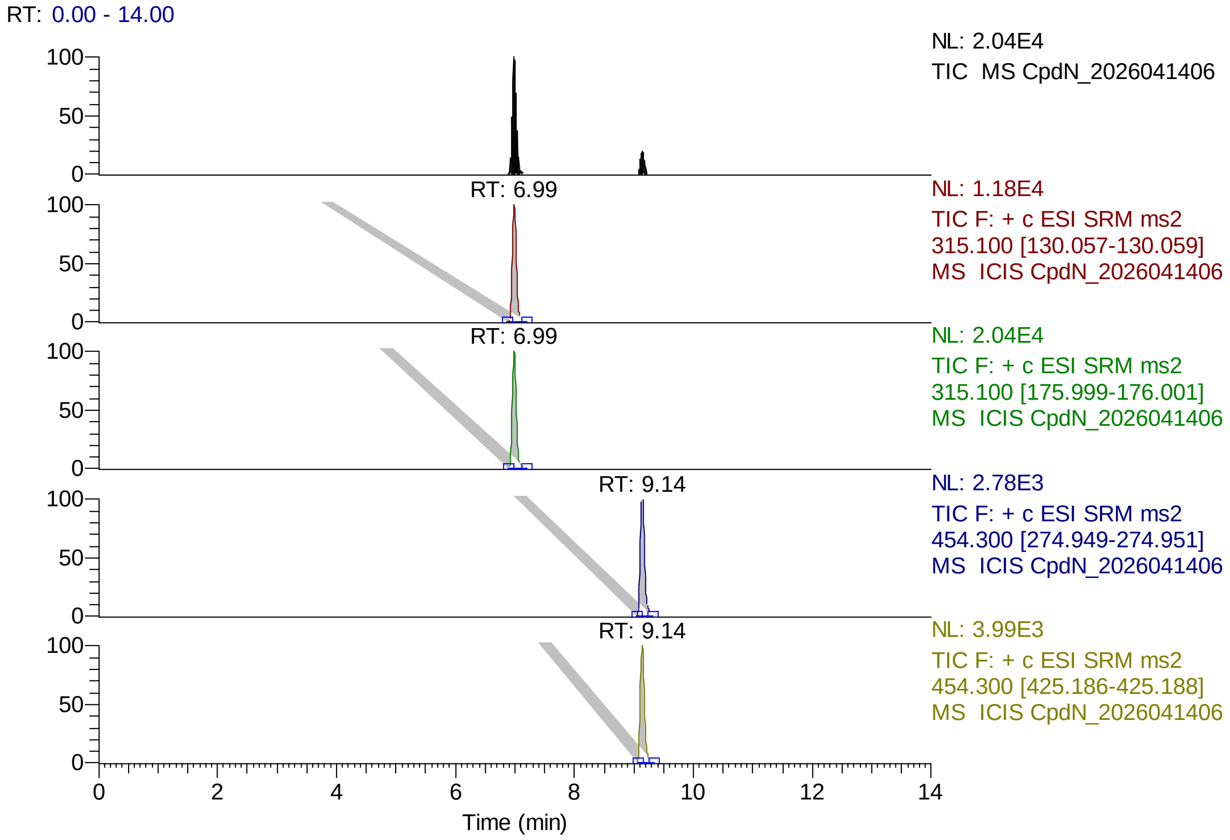
RT: 0.00 - 14.00
NL: 2.04E4
TIC MS CpdN_2026041406
RT: 6.99
NL: 1.18E4
TIC F: + c ESI SRM ms2 315.100 [130.057-130.059] MS ICIS CpdN_2026041406
RT: 6.99
NL: 2.04E4
TIC F: + c ESI SRM ms2 315.100 [175.999-176.001] MS ICIS CpdN_2026041406
RT: 9.14
NL: 2.78E3
TIC F: + c ESI SRM ms2 454.300 [274.949-274.951] MS ICIS CpdN_2026041406
RT: 9.14
NL: 3.99E3
TIC F: + c ESI SRM ms2 454.300 [425.186-425.188] MS ICIS CpdN_2026041406
Time (min)

Supplementary Figure S1. Representative LC–MS/MS chromatograms showing the detection of the internal standard and the analyte netarsudil. A. The internal standard eluted at a retention time of approximately 6.9 min and was monitored in positive electrospray ionization (ESI) selected reaction monitoring (SRM) mode using the m/z 315 → 176 transition. B. The analyte, netarsudil, eluted at approximately 9.14 minutes in the brain of a male wild-type mouse. C. The analyte, netarsudil, eluted at approximately 9.11 minutes in the brain of a female wild-type mouse. D. The analyte, netarsudil, eluted at approximately 9.16 minutes in the plasma of a male wild-type mouse. E. The analyte, netarsudil, eluted at approximately 9.14 minutes in the plasma of a female wild-type mouse. The analyte was detected under the same conditions as the internal standard, using the m/z transition 454 → 425. The distinct retention times and specific SRM transitions demonstrate the selectivity of the analytical method.
